# Hydrogel-Based Biomaterials: A Patent Landscape on Innovation Trends and Patterns

**DOI:** 10.3390/gels11030216

**Published:** 2025-03-20

**Authors:** Ahmed Fatimi, Fouad Damiri, Nada El Arrach, Houria Hemdani, Adina Magdalena Musuc, Mohammed Berrada

**Affiliations:** 1 Chemical Science and Engineering Research Team (ERSIC), Department of Chemistry, Polydisciplinary Faculty of Beni Mellal (FPBM), Sultan Moulay Slimane University (USMS), P.O. Box 592, Mghila Campus, Beni Mellal 23000, Morocco; 2 Laboratory of Biology and Health, Faculty of Sciences Ben M’Sick, University Hassan II of Casablanca, Casablanca 20000, Morocco; fouad.damiri@univh2c.ma (F.D.); nadaarrach@gmail.com (N.E.A.); hemdani.houria@gmail.com (H.H.); mohammed.berrada@univh2c.ma (M.B.); 3 Institute of Physical Chemistry—Ilie Murgulescu, Romanian Academy, 202 Spl. Independentei, 060021 Bucharest, Romania; amusuc@icf.ro

**Keywords:** polymers, hydrogels, biomaterials, innovation, patent landscape

## Abstract

The hydrogel patent landscape is characterized by rapid growth and diverse applications, particularly in the biomedical field. Advances in material science, chemistry, novel manufacturing techniques, and a deeper understanding of biological systems have revolutionized the development of hydrogel-based biomaterials. These innovations have led to enhanced properties and expanded applications, particularly in regenerative medicine, drug delivery, and tissue engineering, positioning hydrogels as a pivotal material in the future of biomedical engineering. In this study, an updated patent landscape for hydrogel-based biomaterials is proposed. By analyzing patent documents, classifications, jurisdictions, and applicants, an overview is provided to characterize key trends and insights. The analysis reveals that hydrogel-related patents are experiencing significant growth, with a strong focus on biomedical applications. Foundational research in hydrogel formation remains dominant, with 96,987 patent documents highlighting advancements in crosslinking techniques, polysaccharide-based materials, and biologically active hydrogels for wound care and tissue regeneration. The United States and China lead in hydrogel-related patent filings, with notable contributions from Europe and a high number of international patents under the Patent Cooperation Treaty (PCT) system, reflecting the global interest in hydrogel technologies. Moreover, emerging innovations include biodegradable hydrogels designed for tissue regeneration, wearable hydrogel-based sensors, and advanced therapeutic applications such as chemoembolization agents and vascular defect treatments. The increasing integration of bioactive elements in hydrogel systems is driving the development of multifunctional biomaterials tailored to specific medical and environmental needs. While this study focuses on patent trends, the alignment between hydrogel research and patenting activities underscores the role of patents in bridging scientific discoveries with industrial applications. Future research could explore patent citation analysis and impact assessments to gain deeper insights into the technological significance of hydrogel-related inventions. Finally, a selection of the top 10 recent active and granted patents in the field of hydrogel-based biomaterials is presented as an illustrative example of innovation in this area and to illustrate cutting-edge innovations.

## 1. Introduction

Recent progress in science and engineering has driven remarkable innovations in hydrogel-based biomaterials, significantly enhancing their potential across a range of biomedical applications, particularly through techniques like 3D bioprinting, which are transforming biomedical applications [1]. These advancements are underpinned by improvements in the fundamental properties of hydrogels, novel synthesis methods, and their expanding roles in areas such as tissue engineering, regenerative medicine, drug delivery, and wound healing [2,3].

Modern hydrogels can now be engineered with superior biocompatibility, biodegradability, and mechanical properties that are tunable for specific biomedical needs [4]. Strategies such as crosslinking and composite manufacturing have been particularly effective in optimizing these properties, allowing hydrogels to perform better in targeted applications [5]. These developments are crucial for creating materials that can mimic the natural extracellular matrix (ECM), making them invaluable in applications requiring integration with biological tissues [6].

The field has seen the emergence of new polymeric materials and hydrogel synthesis techniques, including the development of stimuli-responsive or “smart” hydrogels, such as polysaccharide nanohydrogels [7]. These hydrogels can undergo structural or volume changes in response to external stimuli such as pH, temperature, and electric fields, opening the door to advanced applications such as controlled drug release and adaptable scaffolds for dynamic biological environments [8].

Hydrogels’ ability to mimic the 3D microenvironment of biological tissues has made them an essential tool in tissue engineering [9]. They provide the scaffold for cell proliferation and differentiation, facilitating tissue regeneration [10,11,12]. Furthermore, their use as bioinks in 3D bioprinting technologies has enabled the creation of living tissue structures and even organ prototypes, making hydrogels a cornerstone of regenerative medicine [13,14,15].

Hydrogels are also pivotal in the development of innovative drug delivery systems [7,16]. Their high water content and porosity allow them to encapsulate therapeutic agents and release them in a controlled manner. The creation of smart hydrogels that respond to external stimuli has further advanced this field, enabling more precise and efficient drug delivery mechanisms that enhance treatment outcomes [2,16].

Beyond these key areas, hydrogels are used in a variety of other biomedical applications, including contact lenses, lubricants, and biosensors [17,18,19,20]. The integration of 3D bioprinting technologies with hydrogels allows for the creation of intricate, customized structures tailored for advanced medical applications, such as personalized implants and diagnostic devices [21].

In the literature, the discussion often revolves around the synthesis, properties, and applications of hydrogels [22,23]. This encompasses a wide range of polymers, including both biopolymers and synthetic polymers, which can serve as hydrogel precursors [15,24,25]. By outlining their pros and cons, biopolymer hydrogels generally offer better biocompatibility and biodegradability, while synthetic polymer hydrogels often provide superior mechanical properties and tunability, though they may lack bioactivity without modification (Table 1).

The patent landscape for hydrogel-based biomaterials has seen significant growth and innovation, particularly in applications related to biomedicine, tissue engineering, and 3D bioprinting [26]. A comprehensive analysis identified 46,941 patent documents related to biopolymer-based hydrogels from 1915 to 2023. This indicates a robust interest in hydrogel technologies, especially in the biomedical field [27]. The year 2020 marked a peak in patent filings, particularly for hydrogel-based bioinks used in 3D bioprinting, which began gaining traction around 2004. The United States, China, and South Korea emerged as the leading countries in hydrogel patenting activities [15,21].

In this study, an updated patent landscape for hydrogel-based biomaterials is proposed. By analyzing patent documents, classification, jurisdiction, and applicants, an overview is given to characterize the rapid growth and diverse applications, particularly in the biomedical field. An important part at the end is proposed to demonstrate the innovation trends in this area by synthesizing a selection of recent active and granted patents related to hydrogel-based biomaterials.

**Table 1:** Trade-offs between using biopolymer- and synthetic-polymer-based hydrogels in biomedical applications.

Type	Sub-Type	Hydrogel	Pros	Cons	Ref.
Natural polymers	Proteins	Collagen	Excellent biocompatibility; promotes cell adhesion and tissue regeneration; biodegradable.	Low mechanical strength; rapid degradation; risk of immune response (animal-derived).	[28,29]
		Gelatin	High biocompatibility; promotes cell growth; biodegradable and cost-effective.	Poor mechanical properties; unstable at physiological temperatures.	[30,31]
	Polysaccharides	Alginate	Non-toxic and biocompatible; gelation under mild conditions; ideal for wound healing and drug delivery.	Limited cell adhesion without modification; poor mechanical strength.	[32,33]
		Chitosan	Antimicrobial properties; supports wound healing; biodegradable.	Limited solubility at physiological pH; requires chemical modification for enhanced properties.	[34,35]
Synthetic polymers	Polyethers	Polyethylene glycol (PEG)	Tunable mechanical properties; non-toxic and non-immunogenic; long-term stability.	Lacks inherent biological signals for cell adhesion; non-biodegradable without modification.	[36,37]
		Poly(lactic-co-glycolic acid) (PLGA)	Biodegradable and biocompatible; tunable degradation rate; FDA-approved for medical applications.	Potential acidic by-products during degradation; requires precise control for uniform degradation.	[38,39]
	Acrylic-based	Polyacrylamide	High mechanical strength non-biodegradable, providing stable structure over time adjustable swelling properties.	Non-biodegradable; toxicity concerns for long-term use.	[40]
	Vinyl-based	Polyvinyl alcohol (PVA)	High water content; biocompatible and stable; good mechanical properties for load-bearing applications.	Requires crosslinking for stability; non-degradable in biological environments.	[41,42]

## 2. Methodology

### 2.1. Databases

In this study, we employed a structured patent search strategy using the Lens patent dataset. The search query was formulated as “title:(hydrogel) OR abstract:(hydrogel) OR claim:(hydrogel)”, applying Boolean operators to ensure a comprehensive retrieval of relevant patents. This approach aligns with previously established methodologies, such as the one described by Fatimi (2022) [21], to capture patents explicitly mentioning ‘hydrogel’ in key fields, namely, the Title, Abstract, and Claims. This strategy minimizes the omission of relevant documents while maintaining precision in dataset selection.

The results, as a CSV file, have been filtered to include only published patent documents (i.e., patent application, granted patent, limited patent, patent of addition, amended patent, and amended application) until 15 September 2024 [43]. The Patentscope search service (administered by the World Intellectual Property Organization (WIPO)) was used to define different International Patent Classification (IPC) codes in relation to hydrogel-based biomaterials [44]. Finally, the Google Patents platform was used to collect and download relevant patent documents [45].

### 2.2. Data Collection

Searching in the Title, Abstract, and Claims fields ensures that all relevant patents are captured. By considering all three fields, the search is broadened to include not only patents where the keyword “hydrogel” is central (as indicated in the title) but also those where hydrogels may be discussed in more detail in the abstract or claims, even if the title does not explicitly mention it [21]. For example, some inventions might focus on a broader technology, and hydrogels may only be a component or secondary focus. These patents might not be identified with a title-only search but would still be relevant to the analysis [46,47].

### 2.3. Patent Families

Grouping by simple families (52,120 results) in the combined search filters out duplicate patent applications across different jurisdictions, leading to a more manageable and accurate dataset. The grouped results provide an overview of unique inventions, reducing redundancy from the same invention being filed in multiple countries. While the broader search strategy yields a larger number of results (96,987 ungrouped, 52,120 grouped), it ensures that no significant patent is overlooked, especially in a complex field like hydrogels, where innovations may be spread across various technological and industrial applications. The combination of fields allows for a more robust analysis of trends and innovations (Table 2).

## 3. Patent Analysis

### 3.1. Patent Documents

**Figure 1:**
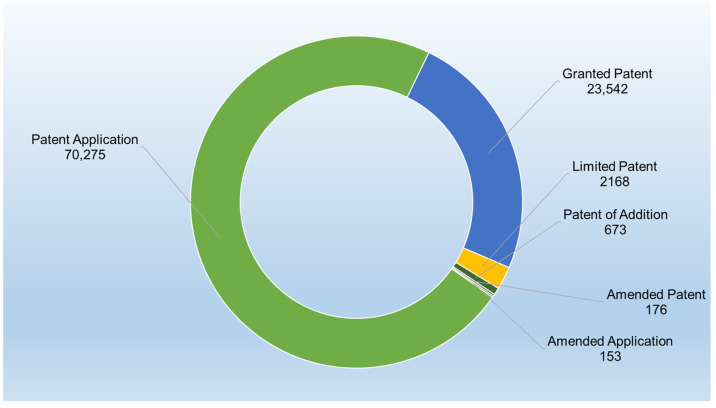
Different types of patent documents and their corresponding counts. This breakdown provides insight into the stages and types of patenting activity in the hydrogel-related technology field.

Until 15 September 2024, a total of 96,987 patent documents were published under different jurisdictions. Figure 1 displays the different types of patent documents (i.e., patent application, granted patent, limited patent, patent of addition, amended patent, and amended application) and their corresponding counts, along with a total of 96,987 patent documents.

The majority of the documents are patent applications, making up 72,275 (around 74.5%) of the total dataset. This suggests that a significant portion of the innovations is in the application stage and has not yet been granted. Granted patents represent 23,542 documents (24.3%). This is the second-largest category, indicating that about a quarter of the patent applications have been successfully processed and approved. There are 2168 limited patents (about 2.2%). These could refer to patents with restricted claims or validity in specific jurisdictions. Their relatively smaller number suggests that fewer inventions are limited in scope. The patent of addition documents’ count is 673 (less than 1%). This category is typically for improvements or modifications to existing patents, indicating that only a small percentage of patents involve subsequent innovations. Finally, amended patents and amended applications make up 176 and 153 documents, respectively. These low numbers (together accounting for less than 0.35%) suggest that only a small proportion of applications or granted patents undergo amendment.

As insights, the data show a clear focus on new patent applications, with a large backlog or ongoing process for many patents still in the application stage. The ratio of granted patents to applications (~33%) reflects the typical attrition in the patenting process, where not all applications result in granted patents. The relatively small number of limited patents, patents of addition, and amended patents/applications indicates that most inventions are either successfully granted as is or are still in the application stage without significant alterations or extensions.

### 3.2. Patent Classification

The IPC system is a hierarchical system used globally to classify patents and utility models according to different areas of technology. The IPC codes are crucial for organizing, retrieving, and analyzing patent documents based on their technological content. The system divides technologies into sections, classes, subclasses, groups, and subgroups to cover all fields of human activity. In the context of analyzing patents, IPC codes are critical for understanding the technological focus of each patent and identifying trends in innovation across different sectors. Table 3, showing document counts for different IPC codes (top 10), provides insights into patenting activity in the hydrogel-related technology field.

The IPC code C08J3/075, with 8865 patent documents, represents patents focused on the formation of macromolecular gels, such as hydrogels, in aqueous media. This high document count suggests that the development of hydrogels and the processes involved in their preparation is foundational to many applications, including medical, industrial, and biotechnological fields. The versatility of hydrogels in absorbing water and maintaining structural integrity makes them critical in these areas.

The code A61L27/52, with 8109 patent documents, highlights patents concerning hydrogels or hydrocolloids used in prostheses or coatings for prostheses. This substantial activity reflects the importance of biocompatible materials in medical devices. Hydrogels’ properties, such as flexibility and moisture retention, make them ideal candidates for prosthetic devices and coatings, contributing to a growing focus on innovation in this area.

A61K9/00, which has 6789 patent documents, deals with medicinal preparations characterized by their physical form, including tablets, gels, and patches. The high level of activity here demonstrates the critical role of hydrogels in the pharmaceutical sector, particularly for their ability to enhance drug delivery systems through controlled release mechanisms.

The A61K9/06 classification, with 5561 patent documents, focuses on ointments and the materials or methods used to produce them. Hydrogels play an essential role in the formulation of topical drug delivery systems, contributing to sustained drug release and hydration in skin applications. This reflects a significant area of hydrogel-related patent activity in pharmaceuticals.

G02B1/04, with 3942 patent documents, covers optical elements made from organic materials, such as plastics. The moderate level of patents indicates steady innovation in contact lens technology, where hydrogels are widely used due to their softness, breathability, and biocompatibility. Hydrogels have revolutionized the contact lens industry, providing comfort and enhanced vision correction.

Similarly, G02C7/04 (with 3924 patent documents) focuses specifically on contact lenses. This classification reflects strong patenting activity in the design and material innovations for vision correction and therapeutic uses of contact lenses. Hydrogels’ importance in providing moisture retention and comfort explains the significant number of patents in this area.

The code A61K47/36, with 3865 patent documents, refers to medicinal preparations that involve polysaccharides or their derivatives, such as alginates and hyaluronic acid. These natural or modified compounds are frequently used in hydrogel formulations to improve drug delivery systems and wound care products. The strong patenting activity reflects the growing use of polysaccharide-based hydrogels in tissue engineering and pharmaceuticals.

The classification C08J3/24, with 3639 patent documents, deals with the crosslinking of macromolecules, a key process in improving the mechanical properties of hydrogels. Crosslinking enhances the strength and stability of hydrogels, making them suitable for a wide range of applications, including medical devices and wound dressings. This highlights the ongoing innovation in improving the durability and functionality of hydrogel materials.

A61L27/54, with 3575 patent documents, refers to biologically active materials used in medical or prosthetic applications. Hydrogels designed to interact with biological systems, often delivering therapeutic substances or promoting tissue regeneration, are of particular interest in this field. The robust patenting activity reflects the role of hydrogels in regenerative medicine, advanced prosthetics, and tissue engineering.

Finally, A61L26/00, with 2943 patent documents, focuses on liquid bandages. Although this is a more specialized area of hydrogel application, it is a growing field within wound care technologies. Liquid bandages, often using hydrogel formulations, provide flexible, protective barriers that promote healing while delivering moisture and therapeutic substances to the wound site.

As key insights, foundational research in hydrogel formation (C08J3/075) leads the field, with nearly 9000 patent documents, underscoring its importance in a wide range of hydrogel applications. Significant patent activity in biomedical uses of hydrogels, particularly for prosthetics (A61L27/52), drug delivery (A61K9/00), and contact lenses (G02C7/04), shows the versatile and growing demand for hydrogels in healthcare technologies. Crosslinking processes (C08J3/24) are essential in ensuring that hydrogels possess the required mechanical strength for their applications, suggesting a continuous focus on material innovation. Polysaccharide-based hydrogels (A61K47/36) and biologically active hydrogels (A61L27/54) are gaining attention, particularly in wound care and tissue regeneration, which are important future directions in hydrogel research. Overall, the data reflect a thriving patent landscape centered on hydrogels’ adaptability in pharmaceuticals, medical devices, and regenerative therapies.

### 3.3. Jurisdictions

**Figure 2:**
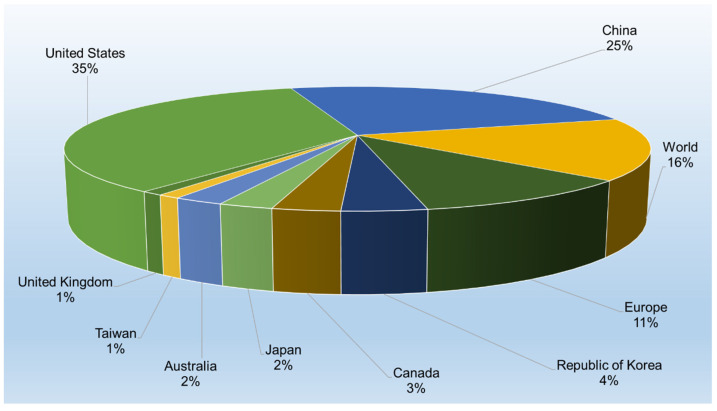
Patenting activity in the hydrogel-related technology field across different jurisdictions (top 10).

Patent jurisdictions refer to the geographical areas or countries where patent protection is sought or granted. Different jurisdictions have their own patent laws and regulations, which can impact how patents are filed, examined, and enforced. Each jurisdiction plays a unique role in the global patent ecosystem, influenced by local laws, market conditions, and innovation activities. Figure 2 displays the patent document percentages for hydrogel-related patents across various jurisdictions (top 10).

With 32,588 patent documents, the United States is the leading jurisdiction for hydrogel patents. This could reflect the country’s large innovation ecosystem, strong intellectual property protections, and significant focus on biomedical and material sciences. China comes in second with 23,265 patent documents, demonstrating its growing prominence in patent filings and technological innovation, especially in fields like healthcare, materials science, and consumer products. World (14,963 patent documents) likely refers to international filings through systems like the Patent Cooperation Treaty (PCT), which allows applicants to seek protection across multiple jurisdictions. This highlights the global importance of hydrogels and the desire to secure international patents. Europe (9764 patent documents) indicates significant innovation within European countries, filed through the European Patent Office (EPO). Hydrogels are clearly of interest across multiple European industries, particularly in healthcare, pharmaceuticals, and material research. The republic of Korea (3563), Canada (2849), Japan (2295), and Australia (2124) all feature prominently, showing that hydrogel innovations are of interest to countries with advanced research and development sectors. Finally, Taiwan (957) and the United Kingdom (951) also contribute smaller but notable shares, reflecting their involvement in specific industries, such as electronics, biotechnology, or healthcare.

As insights, hydrogels are a key area of innovation in many countries, with a strong presence in major innovation hubs (United States, China, and Europe). The high document count in jurisdictions like the United States and China suggests that both countries have substantial hydrogel-related research and industrial applications, while smaller regions like Taiwan and the UK may be more niche players. The significant number of world patent documents underscores the strategic importance of securing patent protection across multiple jurisdictions, emphasizing the global nature of hydrogel technology.

### 3.4. Applicants

**Figure 3:**
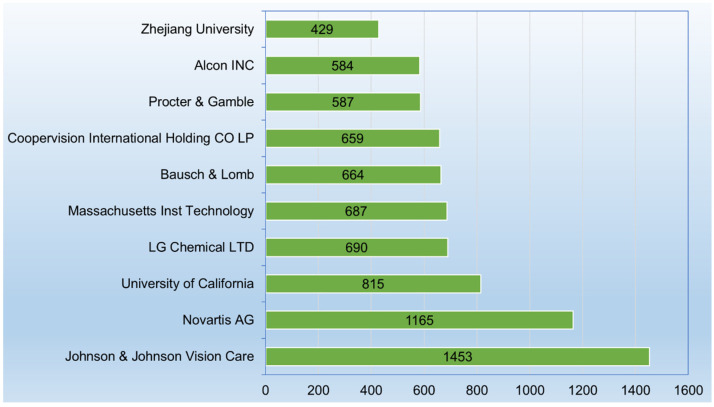
Top 10 patent applicants and their respective patent document counts. This breakdown provides patenting activity in the hydrogel-related technology field as a function of the main types of patent applicants.

A patent applicant is an individual or entity that submits a patent application to seek legal protection for an invention. The applicant is often the person or organization that has developed the invention or holds rights to it. Figure 3 displays the top 10 patent applicants and their respective patent document counts. These data show a strong interest in hydrogel technologies across both corporate and academic sectors, with particular emphasis on their applications in healthcare, vision care, and consumer goods.

Johnson & Johnson Vision Care (Jacksonville, FL, USA) leads with 1453 patent documents, indicating a dominant position in hydrogel-related patents. This likely reflects their extensive R&D in ophthalmic products, such as contact lenses, which frequently use hydrogel materials. Novartis AG (Basel, Switzerland) follows closely with 1165 patent documents. Novartis, a major player in the pharmaceutical and healthcare sectors, likely focuses on hydrogels in drug delivery systems or medical devices.

The University of California (Oakland, CA, USA), with 815 patent documents, and the Massachusetts Institute of Technology (Cambridge, MA, USA), with 687 patent documents, represent strong contributions from academia. These institutions are likely driving fundamental research in hydrogels, covering a wide range of applications, from biomedical engineering to materials science. Zhejiang University (Hangzhou, China), with 429 patent documents, also plays a significant role in hydrogel-related innovation, showcasing China’s growing influence in scientific research and technology development.

As a chemical and technology firm, LG Chemical LTD (Seoul, Republic of Korea), with 690 patent documents, highlights the role of chemical companies in developing hydrogel technologies, possibly in areas like electronics, coatings, and biomedical applications. Procter & Gamble (Cincinnati, OH, USA), with 587 patent documents, and Bausch & Lomb (Laval, QC, Canada), with 664 patent documents, emphasize the importance of hydrogels in consumer products and medical devices, particularly in personal care, vision care, and health-related innovations.

Finally, Coopervision International Holding CO LP (Fareham, England), with 659 patent documents, and Alcon INC (Geneva, Switzerland), with 584 patent documents, are both specialized in eye care, likely focusing on hydrogel formulations for contact lenses and related vision care products.

As insights, the top 10 applicants reflect a mix of corporate (healthcare, chemical, and consumer goods) and academic institutions, indicating the diverse applications of hydrogels across industries and research fields. Healthcare and vision care dominate the dataset, with companies like Johnson & Johnson, Bausch & Lomb, and Coopervision showing substantial innovation in hydrogel materials. Academic institutions are making significant contributions, potentially bridging the gap between fundamental research and industrial applications.

## 4. Recent Patents on Hydrogel-Based Biomaterials

As implications of patent trends, recent progress in science and engineering has significantly advanced innovation in hydrogel-based biomaterials. The latest achievements in hydrogel-based biomaterials focus generally on their development and prospects in medical applications such as injectable biomaterials [48].

The patent landscape for hydrogel-based biomaterials has seen significant developments in recent years, reflecting the growing interest and innovation in this field, as demonstrated by inventions and patents. To demonstrate the innovation trends in hydrogel-based biomaterials, a selection of recent active and granted patents related to hydrogel-based biomaterials is proposed here (Table 4). This selection of the top 10 recent active and granted patents in the field of hydrogel-based biomaterials is presented as an illustrative example of innovation in this area. The selection is based on three specific criteria: the legal status as active, the patent document type as granted patents, and the most recent publication dates, up to 15 September 2024.

**Figure 4:**
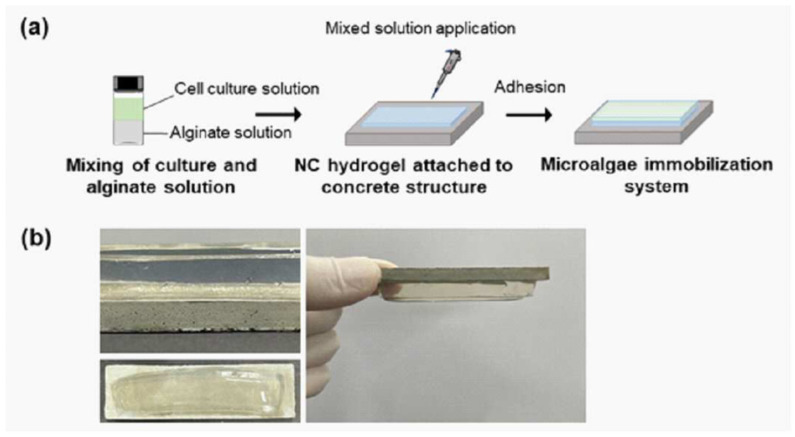
Scheme of the microalgae immobilization process of the complex nanocomposite (NC) hydrogel of the invention: (**a**) the parent formula of the microalgae immobilization process; (**b**) concrete adhesion of alginate gel and complex hydrogel with fixed algae. (Printed from the patent KR102707093B1 [49]).

Patent KR102707093B1 encloses an invention that focuses on the development of complex hydrogels with enhanced adhesion and mechanical properties, particularly suitable for supporting the proliferation, immobilization, and storage of aquatic organisms. According to the inventors Na and Kim (2024) [49], the manufacturing process begins with preparing a composition by mixing monomers, crosslinking agents, initiators, catalysts, and distilled water. Figure 4 shows the microalgae immobilization process of the complex hydrogel of the invention [49]. This mixture undergoes a reaction to form nanocomposite hydrogels, which are then immersed in an aqueous metal salt solution. After immersion, the nanocomposite hydrogel is dried, and an alginate solution is applied to its surface. Finally, the alginate solution reacts with the dried hydrogel, resulting in the production of complex hydrogels with improved functionality for aquatic applications [49].

**Figure 5:**
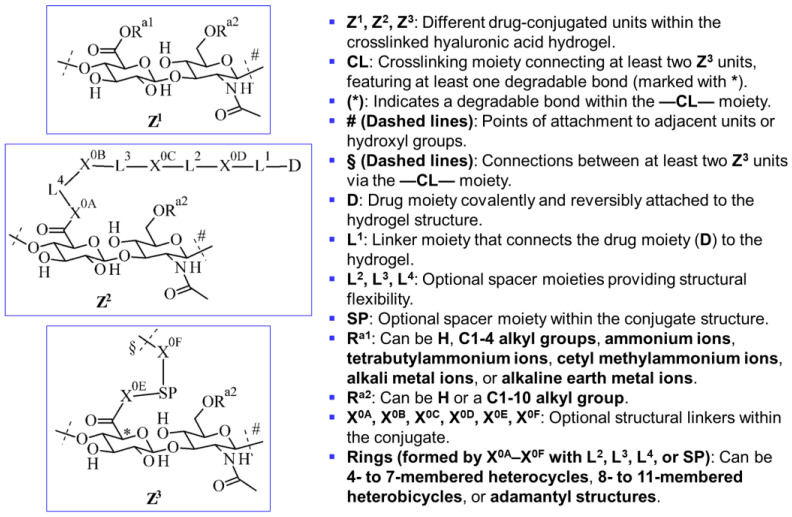
A conjugate structure comprising crosslinked hyaluronic acid strands with covalently and reversibly conjugated drug moieties, represented by units Z^1^, Z^2^, and Z^3^ (adapted from the patent AU2019348440B2 [50]).

The invention described in patent AU2019348440B2 relates to crosslinked hyaluronic acid hydrogel conjugates with covalently and reversibly attached drug moieties (Z^1^, Z^2^, and Z^3^). Stark et al. (2024) [50] modified the hyaluronic acid to a specific degree, which contains degradable crosslinked components. These conjugates are intended for use in the biomedical field as medicaments, with potential applications in the diagnosis, prevention, and treatment of various diseases [50]. As illustrated in Figure 5, the conjugate structure consists of crosslinked hyaluronic acid strands with multiple drug moieties (D) linked via specialized linker moieties (–L^1^–, –L^2^–, –L^3^–, and –L^4^–). These linkers provide structural flexibility and, in some cases, form heterocyclic or adamantyl ring structures. The reversible covalent attachment ensures controlled drug release. Key structural elements include:

- Degradable crosslinked components (—CL—), ensuring controlled breakdown.
- Spacer moieties (—SP—, L-moieties, X-linkages), enhancing molecular flexibility.
- Drug moieties (—D), covalently attached through –L^1^– for controlled release.

This invented design enhances the biocompatibility and functionality of hydrogel-based drug delivery systems. More technical details, including molecular variations and specific interactions, are detailed by Stark et al. (2024) and provided in the granted patent [50].

The invention of patent EP4269458B1 provides a high-oxygen-permeability silicone hydrogel composition, designed for use in contact lenses, along with a method for manufacturing these lenses. Chen et al. (2024) [51] proposed a composition that includes a first and second silicone polymer, at least one hydrophilic monomer, a crosslinker, an initiator, and a solvent. According to the invention, more than one kind of hydrophilic monomer can be used at a time, and the high-oxygen-permeability silicone hydrogel composition can also be used with an additional third silicone polymer to make contact lenses (Table 5) [51]. Analyzing the experimental findings, based on the physical properties such as the water content of the contact lens, the tensile modulus, and the oxygen permeability, the invented hydrogels meet the market demand for contact lenses. By adjusting the mixing ratio of the silicone polymers and hydrophilic monomers, the invention enhances the oxygen permeability of the contact lenses while also expanding the design possibilities for silicone hydrogel lenses [51].

The invention, enclosed in patent EP3870365B1, relates to a system featuring a microfluidic device designed to provide mechanical stimulation to a material. The device developed by Le Gac et al. (2024) [52] consists of a hosting chamber, a pressure array, and an elastic membrane. The hosting chamber holds different materials, such as different hydrogels, with different compositions, especially providing different materials in a layered manner (e.g., hydrogel comprising beads), while the membrane, positioned between the pressure array and the chamber, is subjected to pressure from the array [52]. The pressure array includes multiple chambers that independently apply pressure to the membrane. Two adjacent pressure chambers share a chamber separator, and the membrane’s position relative to this separator varies depending on the pressures applied by the adjacent chambers. According to the invention, the presence of different materials in the microfluidic device may be beneficial for specific model systems, for example, to simulate the successive cellular layers present in cartilage [52].

**Figure 6:**
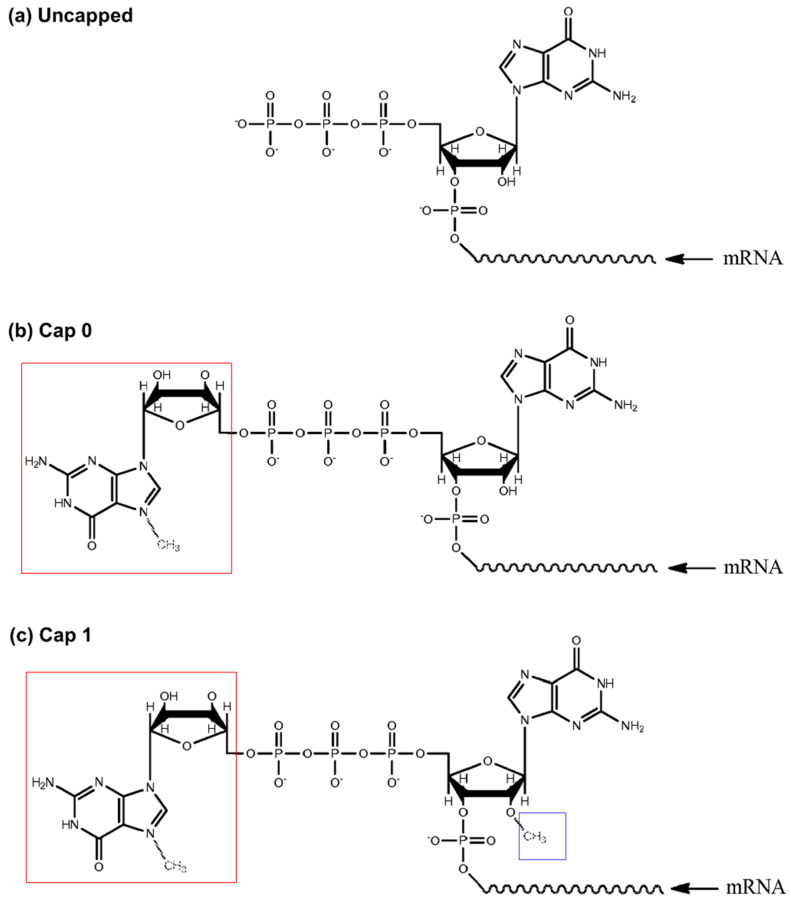
Examples of mRNA-capped structures and an uncapped structure present in various embodiments of the invention: (**a**) uncapped mRNA may be present in a sample (i.e., as a result of incomplete capping in an in vitro transcription reaction) and/or may be used as a control to quantitatively measure the level of uncapped species in a sample; (**b**) Cap0 structures lack a 2′-O-methyl residue of the ribose attached to bases 1 and 2; (**c**) Cap1 structures have a 2′-O-methyl residue at base 1 (adapted from patent EP3495505B1 [53]).

According to patent EP3495505B1, the invention introduces methods for quantifying messenger ribonucleic acid (mRNA) capping (Cap) efficiency, particularly for mRNA synthesized in vitro. According to Heartlein et al. (2024) [53], these methods involve preparing an mRNA sample containing both capped and uncapped mRNA, introducing a cap-specific binding substance under conditions that allow it to form a complex with the capped mRNA, and then quantitatively measuring the amount of this complex in comparison to a control. This process enables the precise determination of mRNA capping efficiency (Figure 6) [53]. In this invention, hydrogels play a crucial role as one of the substrates for capturing mRNA, contributing to the functionality and versatility of the method. Hydrogels, with their high water content, porous structure, and biocompatibility, offer a suitable environment for binding and retaining biomolecules. This makes them particularly advantageous for bioassays, diagnostics, and other applications where gentle handling and high capture efficiency of delicate biomolecules like mRNA are required. The use of hydrogels as a substrate in this invention highlights their role not only as a medium for molecular capture but also as a versatile tool in advancing biotechnological applications, offering advantages over traditional substrates in terms of flexibility, biocompatibility, and tunable properties [53].

Patent EP3630078B1 claims an invention that describes a chemoembolization agent consisting of an embolizing particle or microsphere, an encapsulating agent attached to the particle through ionic or non-covalent interactions, and one or more therapeutic agents within the encapsulating agent. Each therapeutic agent is either uncharged, weakly charged, or has low solubility in aqueous media at physiological pH. Owing to the inventors Na et al. (2024) [54], the encapsulating agent is designed to release the therapeutic agent. The disclosure also covers the uses of this chemoembolization agent and methods for its production. The invention claims that preferred embodiments of the chemoembolization agent include compositions that select embolizing particles or microspheres from sulfonate-modified PVA hydrogel beads, carboxyl-modified PVA-co-sodium acrylate beads, and sulfonate-modified hydrogels [54]. In certain embodiments, the embolizing particle is a hydrogel microsphere coated with an inorganic perfluorinated polymer. For proof of concept, biocompatible PVA hydrogel beads were used, with diameters ranging from 100 to 300 microns. The undiluted solution concentration of these beads was 200,000 per 2 mL. Due to their hydrophilic nature, the biocompatible beads required handling in an aqueous medium. To enable ionic association with cationic encapsulating agents, these beads were modified with negatively charged alkyl sulfonate groups [54].

**Figure 7:**
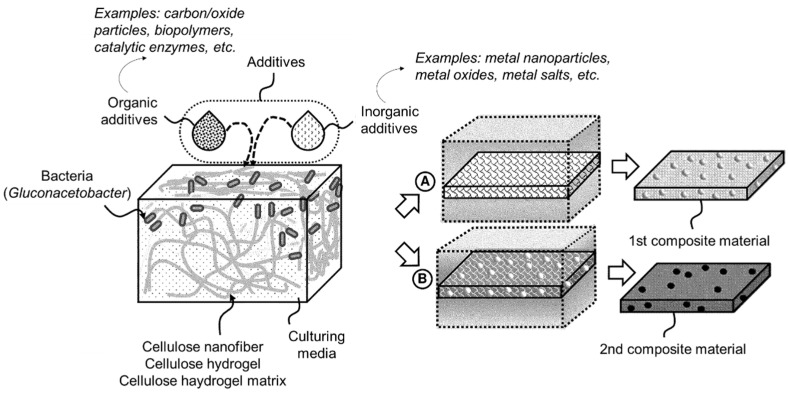
Schematic diagram illustrating a workflow for the production of bacterial cellulose composite materials using a Gluconacetobacter-based hydrogel matrix (adapted from patent US12084701B2 [55]).

Patent US12084701B2 describes an invention that relates to a biofabrication method for producing functionalized cellulose-based composite materials. The invention utilizes a cellulose nanofiber hydrogel produced by bacteria with a framework of bacterial cellulose that offers advantages over plant-derived cellulose due to its nanoscale structure and biofabrication compatibility. The process invented by Terrell and Jahnke (2024) [55] involves providing active cellulose-producing bacteria, such as *Gluconacetobacter*, along with culturing media in a container. Organic or inorganic additives are combined with the bacteria to produce a cellulose hydrogel matrix composed of entangled cellulose nanofibers. The concentration of the additives within the hydrogel matrix is controlled, and the matrix is then exposed to a specific thermal environment to create a biofabricated functional material [55]. Based on the role of the hydrogel, it serves as a biocompatible matrix that allows for the integration of various organic and inorganic additives. These additives can diffuse or bind to the cellulose nanofiber hydrogel structure as it is formed, enabling customization of material properties (e.g., dielectric, electrocatalytic capabilities, etc.). Finally, the post-production steps (e.g., freeze-drying, pyrolysis, etc.) allow for the conversion of the hydrogel into advanced functional materials, such as dehydrated or carbonized composites (Figure 7). Owing to this invention, by integrating additives during the culturing process, the developed hydrogel matrix can yield composite materials with tailored properties for specialized applications, leveraging the bio-friendly and scalable production facilitated by *Gluconacetobacter* [55].

**Figure 8:**
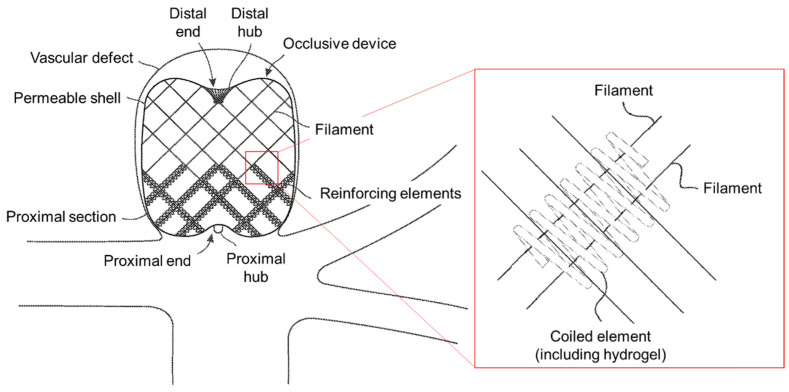
Schematic illustration of the invented device, deployed within an aneurysm, for treatment of a patient’s vasculature that includes coils and hydrogel in a proximal area. The square inset illustrates an example of a configuration of reinforcement elements integrated into a mesh of the device using hydrogels. Hydrogel’s role is dual-purpose: promoting biological integration and enhancing the mechanical properties of the occlusive device for effective vascular occlusion (adapted from patent US12082819B2 [56]).

Patent US12082819B2 relates to an invention of methods for treating a patient’s vasculature, specifically involving a permeable implant. Rangwala et al. (2024) [56] developed an implant that features a radially constrained state for delivery through a catheter and an expanded state for deployment. It is composed of woven elongate filaments and includes a stiffer proximal portion designed to sit at the neck of an aneurysm. This proximal portion may incorporate coils, stiffening elements, or reinforcement elements, either integrated with or woven into the filaments to provide additional structural support (Figure 8) [56]. Hydrogels play an important role in device occlusion and structural reinforcement through this invention. The hydrogel component is incorporated within the coiled elements of the proximal section of the occlusive device to enhance its blood-flow-blocking capabilities. The hydrogel is configured to expand upon exposure to blood, leveraging its reaction to the blood’s pH and aqueous nature. This expansion increases the device’s occlusive effect by occupying more space and creating a more substantial physical barrier within the vessel. Based on different embodiments of the invention, the hydrogel can be applied in several configurations. Firstly, the hydrogel can be coated around the wound wire, forming the coil, allowing it to radially extend from the wire upon expansion. Alternatively, the hydrogel can be situated within the coil’s lumen, enhancing its space-filling properties. Hydrogel may also be selectively placed along specific proximal braid structures, including miniature springs or other reinforcement elements, to promote endothelial growth over the neck of an aneurysm. This re-endothelialization can help close off the aneurysm over time, providing a long-term therapeutic effect [56].

**Figure 9:**
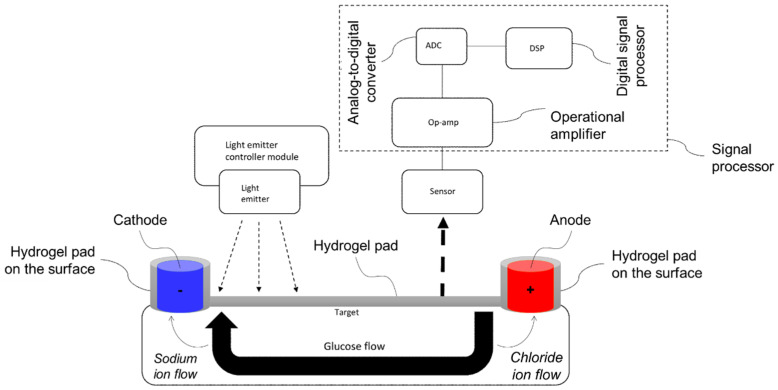
Schematic illustration of the invented noninvasive glucose sensor and continuous glucose-monitoring system. The anode electrode draws negative ions, such as chloride ions, towards it. On the other hand, the cathode electrode draws positive ions, such as sodium ions, towards it. The flow of the sodium ions draws water molecules towards the cathode. However, glucose molecules are also forced, under osmotic pressure, toward the cathode (adapted from patent US12082910B2 [57]).

The invention, through patent US12082910B2, describes systems and methods for using a photoacoustic sensor to estimate analyte concentration levels. It also includes methods for training the sensor to improve accuracy based on the acoustic signals received. Based on the invention claimed by the inventors Zhou et al. (2024) [57], the analyte monitor comprises a light emitter that directs light toward a target, a sensor that detects acoustic waves produced by analyte molecules such as glucose in response to the light, and two electrodes (i.e., cathode and anode), coated and spaced with hydrogel, configured to contact the target. A voltage controller biases the electrodes, while a signal processor estimates the analyte concentration based on the acoustic waves detected by the sensor. In this system, hydrogel plays a key role in the configuration and functionality of the electrodes, which are designed to interface with the target, where the glucose flow is monitored (Figure 9) [57]. In certain embodiments, both electrodes are equipped with hydrogel pads on their surfaces that ensure effective contact with the target surface. This setup allows the hydrogel to enhance the adhesion and conformability of the electrodes to irregularities on the target surface, leading to a more stable and reliable connection. More specifically, the hydrogel pads provide an ionically conductive interface between the electrodes and the target surface. This ion-conductive property supports efficient signal transmission by maintaining consistent electrical contact with the target, critical in applications where bioelectric signals or biochemical changes, like glucose levels, need to be accurately detected. Furthermore, in other preferred embodiments, the electrodes are spaced at a distance of less than 3 cm. This configuration, combined with the hydrogel’s ability to facilitate charge conduction, minimizes impedance and improves the sensitivity of the glucose flow measurement. By incorporating hydrogel pads on the electrodes, this invention achieves improved sensitivity and reliability in measuring glucose flow. The hydrogel’s ability to provide both structural conformity and enhanced ionic conductivity between the electrodes and the target surface is central to this design’s effectiveness [57].

The invention of patent US12083245B2 introduces compositions and methods for wound healing and tissue regeneration. According to inventors Murphy et al. (2024) [58], the compositions include amniotic membrane powder and, in some cases, scaffold. Specifically, the composition contains amniotic membrane powder with a total released protein content ranging from 30 to 500 mg/g. It also features a hydrogel matrix composed of hyaluronic acid and gelatin, crosslinked via a PEG-based crosslinker through maleimide-thiol bonds. In some cases, additional hydrogel matrices based on synthetic polymers such as (meth)acrylate, poly(ethylene oxide) (PEO), poly(propylene oxide) (PPO), and (PPO–PEO–PPO) triblock copolymers (Pluronics) are proposed (Table 6). The methods involve applying these compositions to a subject to promote wound healing and tissue regeneration [58]. The hydrogel-based system allows for easy application as it comes pre-packaged in a dual-chamber syringe that is simple to use and requires minimal preparation. This design supports sterile application using only one hand, making it particularly convenient in clinical settings. The hydrogel’s role as a carrier medium also helps deliver amniotic powder effectively to the wound site, allowing for optimal contact with the healing tissue. In terms of wound-healing quality, the hydrogel-amnion powder combination significantly accelerates healing through faster wound closure and epithelialization rates. Studies show that amniotic powder, when combined with the hydrogel, prevents wound contraction better than the hydrogel alone. Treatments incorporating amniotic powder were found to enhance wound closure through improved re-epithelialization, with only slight delays when powder was diluted in the hydrogel. This controlled release of bioactive factors helps promote rapid and complete healing, creating a new epidermis and dermis that closely resemble healthy skin structures. Histological analysis further highlights the advantage of using the hydrogel in combination with amniotic powder by showing improved skin quality in treated wounds. Amnion powder with hydrogel led to the development of mature, healthy skin-like tissue with organized collagen fibers and elastin that mimic the structure of intact skin. This ECM composition, characterized by intertwined, well-organized fibers, is essential for the strength and longevity of the healed area. The hydrogel matrix facilitates this organized tissue formation, supporting the delivery and effectiveness of the amniotic membrane powder’s bioactive factors. This structure and ECM maturity are significant for the successful and lasting repair of damaged tissue, which untreated wounds and those treated with hydrogel alone failed to achieve. Mechanically, the hydrogel and amniotic powder combination positively impacts the biomechanical properties of the healing tissue. Though there were slight differences in compressive strength, the data indicate that tissues treated with hydrogel and amnion powder demonstrate higher elasticity, as shown by improved Young’s modulus values compared to untreated wounds. This elasticity is a promising feature for skin regeneration as it better replicates the flexibility and resilience of healthy tissue. The hydrogel matrix helps maintain the elasticity of the healing tissue while facilitating a strong, organized collagen structure that allows the regenerated skin to deform and rebound similarly to natural skin. Together, the hydrogel’s structural support and amnion powder’s bioactivity create a wound environment that fosters not only healing but also the formation of functionally and aesthetically similar tissue to natural, healthy skin [58].

## 5. Limitations and Future Research Directions

Our analysis provides a comprehensive patent landscape on hydrogel innovations, focusing on trends in patent filing, assignees, and technological advancements. While this study does not perform a direct comparison with hydrogel research in the scientific literature, notable research trends—such as developments in biomimetic, self-healing, and stimuli-responsive hydrogels—align with observed patenting activities. It is essential to highlight that patentability requires inventions to meet three fundamental criteria: novelty, inventiveness, and industrial applicability. If an innovation has already been published in the scientific literature, it cannot be patented. Therefore, patented hydrogel inventions not only emerge from scientific advancements but also contribute to innovation by translating research into practical applications. This reinforces the role of patents in bridging scientific discoveries and technological progress.

While this study provides a quantitative analysis of hydrogel patent activity, it does not assess the quality, originality, or technological significance of individual patents. Evaluating these aspects would require a qualitative approach, such as patent citation analysis, expert assessments, or impact studies. Future research could further explore these dimensions to provide deeper insights into the technological significance and innovation potential of hydrogel-related patents, as well as the interplay between scientific research and patenting dynamics in this field.

## 6. Conclusions

The analysis of patent data through this study provides a clear picture of the most prominent areas of research and patenting activity around hydrogels, with a strong focus on healthcare applications, drug delivery systems, and medical devices. The analysis of hydrogel-based biomaterial patents highlights a dynamic and evolving field, with a large proportion of applications still in process and a typical grant rate of around 33%. Data concerning patent classification reveal a thriving patent landscape in hydrogel-related technologies, particularly in medical, pharmaceutical, and optical applications. The wide range of uses, from drug delivery systems to prosthetics and contact lenses, demonstrates hydrogels’ versatility and continued innovation across multiple fields. The patenting activity reflects ongoing advancements in the preparation, crosslinking, and functionalization of hydrogels to meet the diverse needs of modern technology and healthcare. Based on the jurisdiction data provided, the United States and China are the leaders in hydrogel-related patents, with substantial contributions from Europe and significant, though smaller, activity from other regions. The high number of global patents under the PCT suggests robust international interest and cross-border innovation in hydrogel technologies. These data highlight the global landscape of hydrogel-related innovations, showing where most of the research and development efforts are concentrated. The involvement of both corporate and academic entities, especially in healthcare, underscores the broad applicability of hydrogels across various industries and the increasing importance of bridging fundamental research with practical, industrial solutions.

This review of recent patents highlights key innovation trends in hydrogel-based biomaterials, underscoring advances across diverse applications. This review primarily focuses on the medical and healthcare applications of hydrogels as they represent the dominant area of patenting activity. While hydrogels are also utilized in food science, agriculture, environmental applications, and water purification, these fields exhibit comparatively lower patenting activity, as evidenced by the patent classification data in Table 3.

The selected patents reflect a strong focus on functionality and versatility in hydrogels, particularly those designed for biomedical, aquatic, and wearable applications. For instance, a hybrid hydrogel developed for propagating aquatic organisms showcases adaptability for environmental and biological applications, while degradable hyaluronic acid hydrogels emphasize biocompatibility and controlled degradation—a critical feature in tissue engineering and drug delivery.

In wearable and implantable technology, patents on high-oxygen-permeability silicone hydrogel compositions and miniaturized non-invasive glucose sensors demonstrate efforts to improve patient comfort and data accuracy in continuous-monitoring devices. Additionally, a microfluidic device for material stimulation and a cap efficiency assessment for mRNA indicate advanced material engineering for precise, responsive biomaterial functions in medical diagnostics and biomanufacturing.

Further patents reveal progress in biofabrication and therapeutic applications. Bacterial cellulose scaffolds in advanced functional materials, chemoembolization agents, and filamentary devices for vascular defect treatment exemplify how bioactive scaffolded hydrogels are tailored for targeted therapeutic interventions. Lastly, an amniotic membrane powder hydrogel for wound healing and tissue engineering captures the therapeutic potential of hydrogels in regenerative medicine, exemplifying how the integration of bioactive elements in hydrogel systems drives innovation in healing and tissue reconstruction applications. These patents collectively represent a robust trend toward specialized, multifunctional hydrogel biomaterials designed to meet emerging medical and environmental needs.

## Figures and Tables

**Table 2:** Patent document counts obtained for different patent queries IPC codes (top 10). The data highlight significant patenting activity in the hydrogel-related technology field, offering insights into various stages and types of innovation.

Search Query	Patent Documents	Simple Families
Title: (hydrogel)	34,858	19,506
Abstract: (hydrogel)	54,797	35,832
Claims: (hydrogel)	50,958	24,367
Title: (hydrogel) OR Abstract: (hydrogel) OR Claims: (hydrogel)	96,987	52,120

**Table 3:** Patent document counts for different IPC codes (top 10). The data highlight significant patenting activity in the hydrogel-related technology field, offering insights into various stages and types of innovation.

IPC Code	Definition	Description	Patent Documents
C08J3/075	Macromolecular gels formed using processes in aqueous media	Encompasses patents dealing with the preparation and processing of macromolecular gels (such as hydrogels) using aqueous solutions, with emphasis on innovations in gel formation methods.	8865
A61L27/52	Hydrogels or hydrocolloids used in prostheses or for coating prostheses	Focuses on innovations in hydrogels or hydrocolloids for medical devices and prosthetic coatings, emphasizing biocompatibility and desirable properties for medical applications.	8109
A61K9/00	Medicinal preparations characterized by their special physical form	Focuses on pharmaceutical formulations where the physical form (e.g., tablets, gels, capsules, patches, etc.) is crucial for optimizing medicine delivery, absorption, or administration.	6789
A61K9/06	Ointments and bases for ointments, including apparatus for making them	Focuses on ointments as a type of medicinal preparation, including formulation bases and the equipment or methods used for producing topical drug delivery forms.	5561
G02B1/04	Optical elements made of organic materials, such as plastics	Covers optical elements (e.g., lenses, prisms, etc.) made from organic materials like plastic, particularly important for contact lenses and lightweight, durable optical devices.	3942
G02C7/04	Contact lenses for the eyes	Focuses on contact lenses characterized by their material or design for vision correction or therapeutic purposes.	3924
A61K47/36	Medicinal preparations involving polysaccharides or their derivatives	Highlights innovations in drug delivery systems, wound care products, or tissue engineering using polysaccharides like gums, alginate, hyaluronic acid, or chitosan.	3865
C08J3/24	Crosslinking of macromolecules	Refers to crosslinking macromolecules, focusing on mechanical aspects and crosslinking agents to enhance material strength, elasticity, and thermal stability, commonly used in hydrogels and vulcanization.	3639
A61L27/54	Biologically active materials used in medical or prosthetic applications	Covers materials designed for biological interaction in regenerative medicine, wound healing, or medical devices, often incorporating hydrogels or biocompatible polymers for therapeutic substance delivery.	3575
A61L26/00	Chemical aspects or materials used for liquid bandages	Focuses on innovations related to the chemical composition and application of liquid bandages for wound care and protection.	2943

**Table 4:** Selection of the top 10 recent active and granted patents related to hydrogel-based biomaterials (until 15 September 2024).

Title	Patent N°	Publication	Family *	Applicants	Ref.
A hybrid hydrogel for propagation of an aquatic organism and a method for manufacturing the same	KR102707093B1	12 September 2024	2S./2Ex.	Hannam University Industry-Academia Cooperation	[49]
Degradable hyaluronic acid hydrogels	AU2019348440B2	12 September 2024	12S./12Ex.	Ascendis Pharma As.	[50]
High-oxygen-permeability silicone hydrogel composition, contact lens made from high-oxygen-permeability silicone hydrogel composition and manufacturing method thereof	EP4269458B1	11 September 2024	6S./6Ex.	Innova Vision Inc.	[51]
Microfluidic device for mechanically stimulating a material	EP3870365B1	11 September 2024	5S./5Ex.	University of Twente	[52]
Quantitative assessment for cap efficiency of messenger RNA	EP3495505B1	11 September 2024	21S./21Ex.	Translate Bio Inc.	[53]
Chemoembolization agents	EP3630078B1	11 September 2024	20S./20Ex.	Bruin Biosciences Inc.; University of California	[54]
Biofabrication of advanced functional materials using bacterial cellulose scaffolds	US12084701B2	10 September 2024	3S./3Ex.	US Army; US Army DEVCOM Army Research Laboratory	[55]
Filamentary devices for treatment of vascular defects	US12082819B2	10 September 2024	11S./11Ex.	MicroVention Inc.	[56]
Miniaturized noninvasive glucose sensor and continuous glucose-monitoring system	US12082910B2	10 September 2024	2S./9Ex.	Medtronic MiniMed Inc.	[57]
Amniotic membrane powder and its use in wound healing and tissue-engineering constructs	US12083245B2	10 September 2024	23S./23Ex.	University Wake Forest Health Sciences	[58]

* Patent families (S.: Simple patent family, Ex.: Extended patent family).

**Table 5:** Examples of different silicone hydrogel compositions with the aforementioned physical properties (water content, tensile modulus, and oxygen permeability) according to embodiments of the invention through patent EP4269458B1 [51].

Compositions and Physical Properties		Formulation					
		1	2	3	4	5	6
Silicone hydrogel compositions	1st silicone polymer	15	20	15	20	10	5
	2nd silicone polymer	30	30	30	30	30	30
	3rd silicone polymer	0	0	0	0	5	5
	1st hydrophilic monomer: HEMA ^1^	35	30	15	5	15	5
	2nd hydrophilic monomer: DMA ^2^	0	0	10	15	10	20
	3rd hydrophilic monomer: NVP ^3^	0	0	10	15	10	15
	Crosslinking agent: EGDMA ^4^	0.8	0.8	0.8	0.8	0.8	0.8
	Photoinitiator: Irgacure 819	1	1	1	1	1	1
	Solvent: n-hexanol	18.2	18.2	18.2	18.2	18.2	18.2
Physical properties	Water content (%)	30.2	26.6	36.1	33.5	38.3	42.3
	Tensile modulus (MPa)	1.1	1.5	0.9	1.2	0.8	0.6
	Oxygen permeability (barrer)	90	105	93	115	90	84

^1^ HEMA: 2-hydroxyethyl methacrylate; ^2^ DMA: N,N-dimethylacrylamide; ^3^ NVP: N-vinylpyrrolidone; ^4^ EGDMA: Ethylene glycol di(meth)acrylate.

**Table 6:** Key claims according to the invention through patent US12083245B2 [58].

Claim	Total Protein Content (mg/g)	Elastin Content (mg/g)	Collagen Content (mg/g)	Glycosaminoglycans Content (mg/g)	TSP-1 ^1^ Content (μg/g)	PTX-3 ^2^ Content (μg/g)	TSG-6 ^3^ Content (ng/g)	Crosslinked Hydrogel Matrix
1	30–500	—	—	—	—	—	—	Hyaluronic acid crosslinked to gelatin via PEG ^4^, maleimide-thiol
2	50–250	—	—	—	—	—	—	Same as Claim 1
3	30–500	4–100	—	—	—	—	—	Same as Claim 1
4	30–500	—	10–800	—	—	—	—	Same as Claim 1
5	30–500	—	—	0.1–5	—	—	—	Same as Claim 1
6	30–500	—	—	—	30–1000	—	—	Same as Claim 1
7	30–500	—	—	—	—	0.1–50	—	Same as Claim 1
8	30–500	—	—	—	—	—	< 1.5	Same as Claim 1
9	30–500	—	—	—	—	—	—	PEGMal ^5^
10	30–500	—	—	—	—	—	—	Methacrylate, PEO ^6^, PPO ^7^, Pluronics ^8^
11	30–500	—	10–600	—	—	—	—	Same as Claim 1
12	—	4–100	—	—	—	—	—	Same as Claim 1
13	—	5–60	—	—	—	—	—	Same as Claim 1
14	—	4–100	10–800	—	—	—	—	Same as Claim 1
15	—	4–100	—	0.1–5	—	—	—	Same as Claim 1
16	—	4–100	—	—	30–1000	—	—	Same as Claim 1
17	—	4–100	—	—	—	0.1–50	—	Same as Claim 1
18	—	4–100	—	—	—	—	<1.5	Same as Claim 1
19	—	4–100	10–600	—	—	—	—	Same as Claim 1
20	30–500	4–100	10–800	0.1–5	—	—	—	Same as Claim 1
21	30–500	4–100	10–800	0.1–5	30–1000	—	—	Same as Claim 1
22	30–500	4–100	10–800	0.1–5	—	0.1–50	—	Same as Claim 1
23	30–500	4–100	10–800	0.1–5	—	—	<1.5	Same as Claim 1
24	30–500	4–100	10–800	0.1–5	30–1000	0.1–50	<1.5	Same as Claim 1

^1^ TSP-1: Thrombospondin-1; ^2^ PTX-3: Pentraxin 3; ^3^ TSG-6: Tumor necrosis factor-stimulated gene 6; ^4^ PEG: Polyethylene glycol; ^5^ PEGMal: PEG based crosslinker with maleimide functional groups; ^6^ PEO: Poly(ethylene oxide); ^7^ PPO: Poly(propylene oxide); ^8^ Pluronics: (PPO–PEO–PPO) triblock copolymers.

## Data Availability

The data presented in this study are available within the content of this article.

## Abbreviations

The following abbreviations are used in this manuscript:

Cap	Capping
DMA	N,N-dimethylacrylamide
ECM	Extracellular matrix
EGDMA	Ethylene glycol di(meth)acrylate
EPO	European Patent Office
Ex	Extended patent family
FDA	U.S. Food and Drug Administration
HEMA	2-hydroxyethyl methacrylate
IPC	International Patent Classification
mRNA	Messenger ribonucleic acid
NC	Nanocomposite
NVP	N-vinylpyrrolidone
PCT	Patent Cooperation Treaty
PEG	Polyethylene glycol
PEG	Polyethylene glycol
PEGMal	PEG-based crosslinker with maleimide functional groups
PEO	Poly(ethylene oxide)
PLGA	Poly(lactic-co-glycolic acid)
Pluronics	(PPO–PEO–PPO) triblock copolymers
PPO	Poly(propylene oxide)
PTX-3	Pentraxin 3
PVA	Polyvinyl alcohol
S	Simple patent family
TSG-6	Tumor necrosis factor-stimulated gene 6
TSP-1	Thrombospondin-1
WIPO	World Intellectual Property Organization
